# Infrapatellar fat pad size and subcutaneous fat in knee osteoarthritis radiographic progression: data from the osteoarthritis initiative

**DOI:** 10.1186/s13075-024-03367-w

**Published:** 2024-07-30

**Authors:** Kwanghoon Lee, Marina Banuls-Mirete, Alecio F. Lombardi, Alexander I.B. Posis, Eric Y. Chang, Nancy E. Lane, Monica Guma

**Affiliations:** 1 Department of Medicine, University of California, San Diego, 9500 Gilman Drive MC 0663, La Jolla, CA 92093-0663 USA; 2 Department of Internal Medicine, Dongguk University Ilsan Hospital, Goyang, Korea; 3 Department of Radiology, University of California, San Diego, La Jolla, CA USA; 4 Herbert Wertheim School of Public Health and Human Longevity Science, University of California San Diego, La Jolla, CA USA; 5 School of Public Health, San Diego State University, San Diego, CA USA; 6 Radiology Service, VA San Diego Healthcare System, San Diego, USA; 7 Department of Medicine, University of California, Davis, Sacramento, CA USA

**Keywords:** Infrapatellar fat pad size, Subcutaneous fat, OAI, Knee OA radiographic progression, Mediation analysis

## Abstract

**Objectives:**

Adipose tissue has been associated with knee osteoarthritis (KOA) pathogenesis, but the longitudinal changes in adipose tissue with KOA progression have not been carefully evaluated. This study aimed to determine if longitudinal changes of systemic and local adipose tissue is associated with radiographic progression of KOA.

**Methods:**

This case-control study used data from the Osteoarthritis Initiative (OAI) and included 315 cases (all the right knees with a minimum of Kellgren-Lawrence score (KL) of 0 and an increase of ≥ 1 KL from baseline to 48 months) and 315 controls matched by age, sex, race, and baseline KL. Cross sectional area of IPFP (IPFP CSA) and subcutaneous adipose tissue around the distal thigh (SCAT*thigh)* were measured using MRI images at baseline and 24 months. Conditional logistic regression models were fitted to estimate associations of obesity markers, IPFP CSA, and SCAT*thigh* with radiographic KOA progression. Mediation analysis was used to assess whether IPFP CSA or SCAT*thigh* mediates the relationships between baseline BMI and radiographic KOA progression.

**Results:**

24-month changes of IPFP CSA (ΔIPFP CSA) and SCAT*thigh* (ΔSCAT*thigh*) were significantly greater in cases compared to controls, whereas Δ BMI and Δ abdominal circumference were similar in both groups during follow-up. Adjusted ORs for radiographic KOA progression were 9.299, 95% CI (5.357–16.141) per 1 SD increase of Δ IPFP CSA and 1.646, 95% CI (1.288–2.103) per 1 SD increase of Δ SCAT*thigh*. ΔIPFP CSA mediated the association between baseline BMI and radiographic KOA progression (87%).

**Conclusions:**

Subjects with radiographic progression of KOA, had significant increases in IPFP CSA and subcutaneous adipose tissue while BMI and abdominal circumference remained stable. Additional studies are needed to confirm these associations.

**Supplementary Information:**

The online version contains supplementary material available at 10.1186/s13075-024-03367-w.

## Introduction

Obesity is one of the strongest risk factors of knee osteoarthritis (KOA) [1]. Although increased mechanical load over the knee joint may explain much of the association between obesity and KOA pathogenesis, various adipokines and inflammatory cytokines secreted by the adipose tissue [2], which are involved in cartilage degradation, synovial inflammation and bone erosion [3, 4], may also contribute to KOA pathogenesis.

Various forms of adipose tissue have been studied for their association with the pathogenesis of KOA. The greater size and volume of systemic adipose tissues such as subcutaneous, visceral, and intermuscular fat have been reported to be associated with KOA symptoms and incidence [5–7]. Infrapatellar fat pad (IPFP) is a type of local adipose tissue in the knee joint that is anatomically close to the synovium and the cartilage. While there seems to be a consensus about the role of IPFP (Hoffa’s) synovitis in KOA, there are conflicting reports on the association of IPFP size with KOA progression. Some studies have reported a protective association of IPFP size with cartilage volume and other structural abnormalities [8, 9]. However, other studies have described an association with KOA progression [10, 11], considering it a potent source of proinflammatory- and matrix degrading mediators [12], and critical in the pathogenesis of KOA [13]. Abnormal signal intensity within the IPFP observed on MRI has been shown to be associated with knee pain [14] and both incident and progressive radiographic KOA [15].

Although many studies suggest the associations between various types of adipose tissue and KOA, the longitudinal changes of adipose tissue during KOA progression have not been investigated comprehensively and it is uncertain which type of adipose tissue is most strongly associated with KOA progression.

In this study, we aimed to compare the longitudinal changes of different adipose tissue measurements between KOA progressors and their controls and to determine their association with KOA progression and whether these measures mediate the association between obesity and KOA progression.

## Materials and methods

### Study population

This case-control study utilized data from the Osteoarthritis Initiative (OAI) cohort (https://oai.nih.gov), which is sponsored by the US National Institutes of Health (NIH) and fully available in (https://oai.nih.gov), and details have been published elsewhere [16]. Briefly, the OAI is a longitudinal, multicenter study of 4796 participants with or at risk for symptomatic knee OA, aged 45–79 years at enrollment. It aimed at identifying biomarkers of development and progression of symptomatic KOA.

**Fig. 1:**
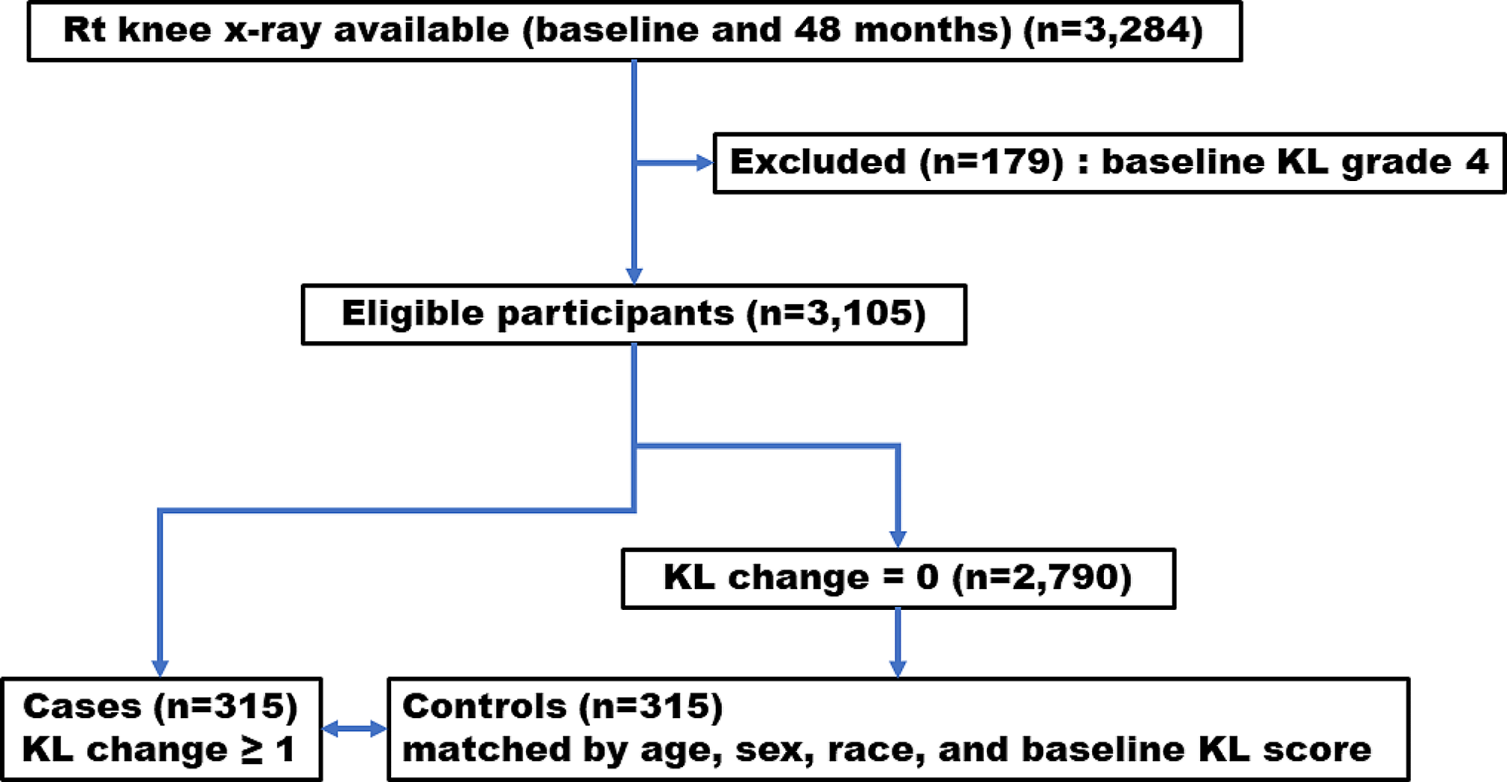
Flow chart of participant selection

We conducted a case-control study, and cases were defined as OAI participants with KOA progression defined as an increase of ≥ 1 Kellgren-Lawrence (KL) score from baseline to 48 months follow-up. We included 3,284 OAI participants with right knee radiographic data available at baseline and 48 months. Participants with a KL score 4 at baseline were excluded. Participants with total knee replacement were classified as KOA progressors if the baseline KL score was < 4 and the progression of KL score was ≥ 1 before surgery. Out of the 3,105 participants, 315 right knees were found to have KL score increase ≥ 1 at 48 months from baseline. 315 controls were randomly selected from those with no KL increase (KL change = 0) by matching by age, sex, race, and baseline KL score. The flow of participant selection is presented in Fig. 1.

From the OAI database, participant characteristics of interest including education level, annual income, history of diabetes, history of injury in the right knee were obtained at baseline, and obesity markers such as body mass index (BMI) and abdominal circumference were obtained both at baseline and at 24 months.

We also performed sensitivity analysis by dividing the participants into incident and progression cohort. Incident cohort was defined as those with baseline KL grade 0 or 1. In this cohort, incident KOA was defined as having KL grade ≥ 2 at 48 months. Progression cohort was defined as those with baseline KL grade ≥ 2. In this cohort, radiographic KOA progression was defined as KL grade change ≥ 1 over 48 months from baseline.

### Imaging of the knee

We used KL grades [17] provided in the OAI database (project number: 15, vendor: Boston University), which was based on the following grade scale: grade 0 = none (absence of osteoarthritis), grade 1 = doubtful (doubtful joint space narrowing), grade 2 = minimal (definite osteophytes and possible joint space narrowing), grade 3 = moderate (multiple osteophytes, definite joint space narrowing and slight sclerosis), and grade 4 = severe (large osteophytes, marked narrowing of joint space, severe sclerosis, deformity of bone ends).

OAI used 3 Tesla (T) knee MRI protocol with the following sequences: coronal intermediate-weighted 2D turbo spin-echo (TSE), sagittal 3D dual-echo in steady state (DESS) with water excitation that can be reformatted on the coronal and axial planes, coronal T1-weighted 3D fast low-angle shot (FLASH), sagittal IW 2D TSE fat-saturated, and sagittal 2D multi-echo spin-echo (MESE). Sagittal intermediate-weighted fat-suppressed turbo spin echo (TR/TE, 3200/30 ms; FA ¼ 180°) and axial reformatted water-excitation dual-echo in steady state (TR/TE, 16.3/4.7 ms; FA ¼ 25°) sequences were also used [18].

Effusion-synovitis and Hoffa-synovitis scores at baseline and 2 years follow-up, which were graded according to the MRI Osteoarthritis Knee Score (MOAKS) [19], were described and published in our recent paper [20]. Cross sectional area (CSA) of IPFP was measured by manually drawing contours around the IPFP boundaries on section-by-section T2-weighted sagittal MR images, using the software program Osiris. Computed single slices were reviewed to find the maximal CSA. The maximal CSA area (cm^2^) was selected to represent the IPFP size (Supplementary Figure S1A). For the measurement of subcutaneous fat thickness around the distal thigh (SCAT*thigh*), the methodology used by Ernandez, et al. [21] was applied. Using the axial fat suppressed T2W images, an axial slide was selected at the level of the uppermost axial plane, which was 7–7.5 cm above the lateral joint line. This slide was divided into four quadrants (anterior medial, anterior lateral, posterior medial, and posterior lateral) and the longest distance from the skin surface to the deep fascia in an imaginary line heading toward the center of the image was measured in each quadrant. The sum of these 4 values was taken as SCAT*thigh* (Supplementary Figure S1B). To ensure intra- and inter- observer reliability, one observer measured both IPFP CSA and SCAT*thigh* on all the MR images with random cross checks (50 cases) performed by a second independent observer. The observer who measured all the MR images repeated the same measurement on randomly selected 20 MR images 6 months later. Root mean square standard deviation (RMS-SD) was used to demonstrate intra-observer and inter-observer reliability. The mean IPFP CSA between observer 1 and 2 was 6.91 ± 1.13 and 6.50 ± 0.97 (RMS SD 1.058). The mean IPFP CSA between observer 1 and observer 1 (6 months later) was 6.91 ± 1.13 and 6.87 ± 1.04 (RMS SD 1.085). The mean SCAT*thigh* between observer 1 and observer 2 was 5.70 ± 2.29 and 6.0 ± 2.51 (RMS SD 2.402). The mean SCAT*thigh* between observer 1 and observer (6 months later) was 5.70 ± 2.29 and 5.85 ± 2.29 (RMS SD 2.29).

### Statistical analysis

The SPSS Statistics version 26 software was used to perform the statistical analyses. Participant characteristics were described using means and standard deviation (SD) or percentages. Comparisons between continuous variables were assessed with Student’s t-test and for categorical variables with chi-square tests. One way analysis of variance (ANOVA) and post hoc Bonferroni comparison were used to compare values among more than 3 groups. Conditional logistic regression analysis was used to determine whether several factors of interest including changes of IPFP CSA over 24 months from baseline (Δ IPFP CSA), changes of SCAT*thigh* over 24 months from baseline (Δ SCAT*thigh*), effusion-synovitis and Hoffa synovitis as well as their change over 24 months are associated with KOA progression (set as an outcome) adjusting for right knee injury (history of undergoing an arthroscopy to repair an injury in the right knee). Δ IPFP CSA and Δ SCAT*thigh* were z-score standardized and the odds ratios (ORs) of KOA progression with these variables set as independent variables were based on each 1 standard deviation (SD) increase of each variable. Multicolinearity was checked for each exposure variable included and all the exposure variables mentioned above had variance inflation factors around 1.

To estimate the proportion of the total effect of BMI on KOA progression mediated by either Δ IPFP CSA or Δ SCAT*thigh*, a mediation analysis was performed through the PROCESS macro for SPSS using 5,000 bootstrap samples (SPSS, Inc., Chicago, IL, USA). Details of the mediation analysis are described elsewhere [22]. Linear and conditional logistic regression models were fit to estimate the total, direct, and indirect effects controlling for prior right knee injury [23]. The model of the mediation analysis in illustrated in Supplementary Figure S2. The total effect is the effect of the BMI (exposure) on KOA progression (outcome) controlling for prior right knee injury. The direct effect is the effect of BMI on KOA progression controlling for either Δ IPFP CSA or Δ SCAT*thigh* and prior right knee injury. The indirect effect is the effect of either Δ IPFP CSA or Δ SCAT*thigh* on KOA progression per one unit increase of BMI controlling for prior right knee injury. The proportion mediated by either Δ IPFP CSA or Δ SCAT*thigh* of the total effect of BMI on KOA progression was calculated based upon these estimates if the natural direct effect and natural indirect effect were in the same direction.

## Results

### Baseline demographics and baseline values of IPFP CSA and SCAT *thigh* of the case and control groups

At baseline, the mean age of the total subjects was 60.9 ± 8.3 years, 70.8% were female, and 87% were White/Caucasian. There were no significant differences in education level, annual income, history of diabetes, and history of injury in the right knee between the cases and the controls. Proportions of each KL grade were 36.8% for grade 0, 30.2% for grade 1, 25.1% for grade 2, and 7.9% for grade 3. BMI (29.1 ± 4.7 kg/m2 vs. 28.2 ± 4.9 kg/m^2^, *P* = 0.014) and abdominal circumference (103.91 ± 12.83 cm vs. 100.95 ± 12.62 cm, *P* = 0.004) were significantly greater in cases than in controls. The baseline characteristics are summarized in Supplementary Table S1. At baseline, while the scores of effusion synovitis (0.71 ± 0.67 vs. 0.41 ± 0.51, *P* < 0.001) and Hoffa synovitis (0.78 ± 0.72 vs. 0.44 ± 0.54, *P* < 0.001) were significantly higher in cases than in controls, IPFP CSA and SCAT*thigh* measurements were similar between groups (Table 1). Of interest, differences in BMI (29.24 ± 5.04 vs. 27.96 ± 5.17, *P* = 0.009) and abdominal circumference (104.05 ± 13.53 vs. 100.10 ± 13.02, *P* = 0.002) between cases and controls were only significant in women (Supplementary Table S2A). Yet, the IPFP CSA and SCAT*thigh* were similar between groups in both men and women, although the SCAT*thigh* tended to be larger in women cases (6.47 ± 1.84 vs. 6.23 ± 1.59, *P* = 0.158, Supplementary Table S2A).

**Table 1:** Baseline values of obesity and imaging markers

Baseline values	Case (*n* = 315, mean ± SD)	Control (*n* = 315, mean ± SD)	*P*
BMI (kg/m^2^)	29.09 ± 4.69	28.1 ± 4.83	0.014
Abdominal circumference (cm)	103.90 ± 12.82	100.95 ± 12.62	0.004
IPFP CSA (cm^2^)	6.51 ± 1.17	6.54 ± 1.17	0.787
SCAT*thigh* (cm^2^)	5.68 ± 2.09	5.50 ± 1.86	0.251
Hoffa synovitis	0.78 ± 0.72	0.44 ± 0.54	< 0.001
Effusion synovitis	0.71 ± 0.67	0.41 ± 0.51	< 0.001

BMI: body mass index, IPFP CSA: cross sectional area of infrapatellar fat pad, SCAT*thigh*: subcutaneous fat assessment around distal thigh, Hoffa synovitis and effusion synovitis were scored based on MOAKS

### Changes of obesity and MRI markers between baseline and 2 years

At 2 years after baseline, Δ IPFP CSA (0.47 ± 0.54 vs. -0.27 ± 0.43, *P* < 0.001) and Δ SCAT*thigh* (0.84 ± 1.36 vs. 0.38 ± 1.19, *P* < 0.001) were significantly greater in cases than in controls, while the changes over 2 years of BMI (ΔBMI) and abdominal circumference (Δ abdominal circumference) were similar in both groups (Table 2). Similarly, the proportions of subjects with 1 standard deviation (SD) increase of IPFP CSA and SCAT*thigh* at 24 months from baseline was greater in cases than in controls (29.4% vs. 0.7%, *P* < 0.001 for IPFP CSA, 13.9% vs. 4.6%, *P* < 0.001 for SCAT*thigh*, Table 3). Sensitivity analysis by sex and BMI category showed that changes in ΔIPFP CSA and ΔSCAT*thigh* were significantly greater in cases than in controls in both men and women, and in the different BMI categories (Supplementary Table S2 B and S2C). Dichotomous analysis comparing the proportions of subjects with increases in BMI (greater than 5% from baseline), abdominal circumference (greater than 5 cm from baseline), SCAT*thigh* (increase greater than 1 SD from baseline), IPFP CSA (increase greater than 1 SD from baseline) and synovitis scores showed similar results. The difference in ΔSCAT*thigh* was remarkable in obese women (Supplementary Table S3). In the group of the cases that progressed at 48 months, 116 (36.8%) of them did not show KL grade progression at 24 months. Subgroup analysis including these 116 cases and the controls revealed that ΔIPFP CSA and Δ SCAT*thigh* were still significantly higher in this group compared to controls. (0.46 ± 0.53 vs. -0.27 ± 0.43, *P* < 0.001 for ΔIPFP CSA and 0.94 ± 1.33 vs. 0.38 ± 1.19, *P* < 0.001 for Δ SCAT*thigh*).

**Table 2:** Changes over 24 months in obesity and imaging markers BMI: body mass index, IPFP CSA: cross sectional area of infrapatellar fat pad, SCAT*thigh*: subcutaneous fat assessment around distal thigh, Δ: changes over 24 months from baseline, Hoffa synovitis and effusion synovitis were scored based on MOAKS

	Case (*n* = 284, mean ± SD)	Control (*n* = 284, mean ± SD)	*P*
**Values at 24 months**			
BMI (kg/m^2^)	29.33 ± 5.07	28.20 ± 4.90	0.006
Abdominal circumference (cm)	105.16 ± 13.43	102.11 ± 12.26	0.004
IPFP CSA (cm^2^)	6.96 ± 1.20	6.23 ± 1.11	< 0.001
SCAT*thigh* (cm^2^)	6.54 ± 2.05	5.87 ± 1.88	< 0.001
Hoffa synovitis	1.25 ± 0.71	0.32 ± 0.47	< 0.001
Effusion synovitis	1.39 ± 0.74	0.37 ± 0.49	< 0.001
**Change over 24 months**			
Δ BMI (kg/m^2^)	0.22 ± 1.76	0.14 ± 1.95	0.633
Δ Abdominal circumference (cm)	1.40 ± 9.32	1.33 ± 9.67	0.934
Δ IPFP CSA (cm^2^)	0.47 ± 0.54	-0.27 ± 0.43	< 0.001
Δ SCAT*thigh* (cm^2^)	0.84 ± 1.36	0.38 ± 1.19	< 0.001
Δ Hoffa synovitis	0.45 ± 0.84	-0.13 ± 0.5	< 0.001
Δ Effusion synovitis	0.67 ± 0.77	-0.06 ± 0.52	< 0.001

**Table 3:** Changes of markers of obesity and synovitis over 24 months (dichotomous analysis)

	All	Case	Control	*P*
BMI increase greater than 5% [*n* = 593, n(%)]	103 (17.4)	51 (17.2)	52 (17.5)	1.000
Abdominal circumference increased greater than 5 cm from baseline [*n* = 587, n(%)]	161 (27.4)	76 (26.0)	85 (28.8)	0.461
ΔSCAT*thigh* greater than 1 SD [*n* = 563, n(%)]	52 (9.2)	39 (13.9)	13 (4.6)	< 0.001
ΔIPFP CSA greater than 1 SD [*n* = 566, n(%)]	85 (15.0)	83 (29.4)	2 (0.7)	< 0.001
Hoffa synovitis worsened [*n* = 566, n(%)]	146 (25.8)	130 (46.1)	16 (5.6)	< 0.001
Effusion synovitis worsened [*n* = 566, n(%)]	192 (33.9)	162 (57.4)	30 (10.6)	< 0.001

BMI: body mass index, IPFP CSA: cross sectional area of infrapatellar fat pad, SCAT*thigh*: subcutaneous fat assessment around

distal thigh, Δ: changes over 24 months from baseline, Hoffa synovitis and effusion synovitis were scored based on MOAKS

### Conditional logistic regression analysis for KOA progression

We used conditional logistic regression models to estimate the odds ratio (OR) for KOA progression of the MRI markers and obesity markers controlling for prior right knee injury. The odds for KOA progression were higher for 1 SD increase of ΔSCAT*thigh* [OR (95% CI) 1.646 (1.288–2.103)] and also higher for 1 SD increase of ΔIPFP CSA [OR (95% CI) 9.299 (5.357–16.141)]. There were no significant associations between KOA progression and baseline values of IPFP CSA [OR (95% CI) 0.977 (0.803–1.188)] and SCAT*thigh* [OR (95% CI) 1.151 (0.924–1.433)]. Among the obesity indicators, baseline BMI [OR (95% CI) 1.286 (1.073–1.542) per every 1 unit (kg/m^2^) increase of BMI] and baseline abdominal circumference [OR (95% CI) 1.297 (1.091–1.541) per every 1 cm increase of abdominal circumference] showed a significant association with KOA progression. Odds for KOA progression was not higher for Δ BMI [OR (95% CI) 1.048 (0.956–1.149)] and for Δ abdominal circumference [OR (95% CI) 1.002 (0.985–1.019)]. Both the baseline values and the changes over 24 months of Hoffa’s synovitis and effusion synovitis were significantly associated with KOA progression (Table 4). Sensitivity analysis by sex showed similar results between men and women except that baseline BMI and baseline abdominal circumference was significantly associated with KOA progression only in women (Supplementary Table S4). Sensitivity analysis by BMI category also showed similar results among normal weight, overweight, and obese group (Supplementary Table S5).

**Table 4:** ORs for radiographic knee OA progression* OR: odds ratio, CI: confidence interval, BMI: body mass index, IPFP CSA: cross sectional area of infrapatellar fat pad, SCAT*thigh*: subcutaneous fat assessment around distal thigh, Δ: changes over 24 months from baseline, Hoffa synovitis and effusion synovitis were scored based on MOAKS * ORs are derived from separate, multivariable conditional logistic regression models

	*OR (95% CI)	*P*
Baseline BMI	1.286 (1.073–1.542)	0.006
Δ BMI	1.091 (0.919–1.295)	0.319
Baseline abdominal circumference	1.297 (1.091–1.541)	0.003
Δ abdominal circumference	1.020 (0.867–1.200)	0.814
Baseline SCAT*thigh*	1.151 (0.924–1.433)	0.209
Δ SCAT*thigh*	1.646 (1.288–2.103)	< 0.001
Baseline IPFP CSA	0.977 (0.803–1.188)	0.814
Δ IPFP CSA	9.299 (5.357–16.141)	< 0.001
Baseline Hoffa’s synovitis	1.691 (1.410–2.027)	< 0.001
Δ Hoffa’s synovitis	2.649 (2.007–3.497)	< 0.001
Baseline effusion synovitis	1.710 (1.416–2.066)	< 0.001
Δ Effusion synovitis	3.752 (2.695–5.225)	< 0.001

### Mediation of the association of baseline BMI with radiographic KOA progression by Δ IPFP CSA or Δ SCAT*thigh*

Our group previously showed that the association of obesity (BMI) with KOA progression was mediated by worsening of the synovitis [20]. In this study, we sought to estimate the proportion mediated of the association between BMI and KOA progression by either of ΔIPFP CSA or ΔSCAT*thigh*. We first sought to determine if there were significant associations between exposure variable (baseline BMI), mediator variable (ΔIPFP CSA or ΔSCAT*thigh*), and outcome variable (KOA progression). From the simple linear regression analysis controlling for prior injury to right knee, baseline BMI was significantly associated with ΔIPFP CSA (F = 6.385, *P* = 0.002 (ANOVA), unstandardized B = 0.031, t = 3.482, *P* < 0.001), but not with ΔSCAT*thigh* (F = 1.117, *P* = 0.328). Conditional logistic regression analysis previously showed that ΔIPFP CSA [OR (95% CI) 9.299 (5.357–16.141) per 1 SD increase], ΔSCAT*thigh* [OR (95% CI) 1.646 (1.288–2.103) per 1 SD increase] and baseline BMI [OR (95% CI) 1.286 (1.073–1.542) per every 1 unit (kg/m^2^) increase of BMI] were significantly associated with radiographic KOA progression. Mediation analysis revealed that Δ IPFP CSA mediated 87% of the association between baseline BMI and radiographic KOA progression. Sensitivity analysis by sex revealed that this mediation was meaningful only in women (83.3%, Table 5). The proportion mediated by Δ SCAT*thigh* could not be estimated because the directions of indirect and direct effect coefficient were different (data not shown).

**Table 5:** Mediation analysis of the effect of obesity indicators change over 2 years on KOA progression mediated by IPFP CSA change over 2 years

Independent variable (X)	Indirect effect coefficient (95% CI)	Direct effect coefficient (95% CI)	Mediation (%)
*All (n = 630)*			
Baseline BMI	0.0721 (0.0340, 0.1188)	0.0107 (-0.0343, 0.0557)	87
*Men only (n = 184)*			
Baseline BMI	0.0499 (-0.0395, 0.1600)	-0.0105 (-0.1132, 0.923)	
*Women only (n = 446)*			
Baseline BMI	0.0825 (0.0350, 0.1424)	0.0165 (-0.0345, 0.0675)	83.3

KOA: knee osteoarthritis, IPFP CSA: cross sectional area of infrapatellar fat pad, CI: confidence interval, BMI: body mass index

### Sensitivity analysis per sub-cohort (incident and progression cohort)

We divided the subjects into incident and progression cohort and did the same analysis as above. Δ IPFP CSA was significantly associated with incident KOA (KL grade ≥ 2 at 48 months) [OR 6.855 (95% CI 2.897–16.220)] in the incident cohort. It was also associated with radiographic KOA progression (KL grade change ≥ 1) [OR 10.576 (95% CI 3.734–29.954)] in the progression cohort. Δ IPFP CSA mediated 27.9% of the association between baseline BMI and incident KOA in the incident cohort and it mediated 97.7% of the association between baseline BMI and radiographic KOA progression in women in the progression cohort. Details of the sub-cohort analysis are shown in Supplementary Tables S6 and S7.

## Discussion

This study describes the longitudinal changes of obesity and MRI measures between KOA progressors and non-KOA progressors. While abdominal circumference and BMI did not change significantly, SCAT*thigh* and IPFP CSA showed significant increases along with the worsening of synovitis in OA progressors. 24-month changes of IPFP CSA mediated 87% of the association of baseline BMI and radiographic KOA progression.

The increase over 2 years in IPFP size (ΔIPFP CSA), representing intra-articular adipose tissue, was strongly associated with KOA progression. There have been controversies on the impact of IPFP size on KOA progression. IPFP size was negatively associated with medial osteophytes, tibial cartilage defects, and joint space narrowing in a study that assessed the associations between IPFP maximum area and KOA features [24]. Another study reported that IPFP volume was positively associated with tibial and patellar cartilage volume, negatively associated with cartilage defects and, number of osteophytes in patients with KOA [8]; suggesting a potentially protective role. In contrast, some studies pointed out that abnormal IPFP could produce proinflammatory cytokines as well as adipokines, and thus might have a detrimental effect on KOA progression [25, 26]. IPFP volume was positively associated with knee pain and inflammation detected by MRI [27] and patients with KOA had higher IPFP volume than asymptomatic controls [28]. Considering that Δ IPFP CSA was associated with radiographic KOA progression, but not the baseline IPFP CSA in this study, it might be the relative increase of IPFP over time, but not its innate size that is implicated in radiographic KOA progression.

The subanalysis involving 116 cases that did not progress radiographically at 24 months showed that Δ IPFP CSA and ΔSCAT*thigh* were significantly greater in cases compared to controls. Similarly, Δ IPFP CSA and ΔSCAT*thigh* were also greater in cases with incident KOA in the incident cohort. This may suggest that the increases of IPFP CSA and SCAT thigh temporally precede the radiographic KOA progression.

Δ IPFP CSA was shown to mediate the association between baseline BMI and radiographic KOA progression. Given that increased IPFP size was reported to be associated with osteophyte area and IPFP was enriched with macrophages as well as various inflammatory cytokines after high fat diet in a mouse model of OA [29], it may be speculated that BMI affects KOA progression by inducing inflammatory conditions in IPFP. This is in line with that obesity affects OA by disrupting immune homeostasis and causing joint inflammation [30].

An increase in SCAT*thigh* was noted in radiographic KOA progressors during follow-up compared to non-progressors. However, BMI remained relatively stable despite the increase of SCAT*thigh*. Sarcopenic obesity, a condition with both sarcopenia and obesity, may explain this gap between BMI and SCAT*thigh*. In addition, there have been several reports on the impact of sarcopenic obesity on KOA. In a cross-sectional study of Korean postmenopausal women, sarcopenic obesity had a greater effect on KOA than obesity without sarcopenia [31]. SCAT was shown to be negatively associated with muscle mass and strength [2]. In addition, thigh SCAT mass has been shown to have stronger association with KOA than thigh muscle mass [7]. Interestingly, sarcopenic obesity was reported to be more strongly associated with KOA in women [32].

In this study, the increase over 2 years of SCAT*thigh* was associated with radiographic KOA progression in both sexes. Previously, SCAT was reported to increase longitudinally only in painful knees [5]. Longitudinal increase of SCAT was also reported to be associated with radiographic KOA progression, but it was not significant in women [7]. In another study, inter muscular adipose tissue but not SCAT was significantly associated with radiographic KOA progression in women [33]. The discrepancy between these previous results and ours could be attributed to the differences in the definition of radiographic KOA progression, methodology to measure SCAT, and study design.

Among the markers of adipose tissue, Δ IPFP CSA had the strongest association with radiographic KOA progression, while baseline abdominal circumference, baseline BMI, and Δ SCAT*thigh* had similar effect sizes smaller than that of Δ IPFP CSA. The latter finding is in line with the study by Culvenor et al. that local or central adiposity had similar associations with incident KOA as BMI [34]. However, the association of IPFP size change over time with KOA progression has not been addressed before. IPFP is in the intra-capsular and extra-synovial space in the knee joint, close to the synovium and other joint structures enabling the intimate interaction between the synovium and IPFP [12]. This may underlie the strong association between changes of IPFP and KOA progression shown in this study.

Although baseline values of BMI and abdominal circumference had significant ORs for radiographic KOA, their changes over 24 months were not significant in this study. It may be that 24 months may not be long enough to see the effect of changes of these variables. Mixed results have been reported regarding the association of increased weight or BMI with structural progression of KOA. Increased weight was reported to be associated with cartilage score progression [35], medial and total cartilage loss [36], and worsening bone marrow lesions [35]. In contrast, another study reported increased weight was not to be associated with cartilage defects or meniscal abnormalities [37].

The criterion of radiographic KOA progression (KL grade change ≥ 1) used in this study may be somewhat vague and weak for determining KOA progression especially when the change is from KL grade 0 to 1. Since many previous studies typically used KL grade change ≥ 1 in the case of progression cohort (established KOA) and KL grade ≥ 2 in the case of incident cohort, we divided the participants into incident cohort and progression cohort and performed the same analysis in each cohort. The similar results from these sub-analyses support the robustness of the results of the current study.

In this study, obesity was more clearly associated with KOA progression in women compared to men. This may be explained by the previous reports about sex differences in KOA. Biomechanically, compared to men, women are more likely to have valgus malalignment [38], have weaker quadriceps muscle [39], unfavorable gait biomechanics [40], and have more compliant ligaments [41]. Accordingly, obese women had a greater risk of KOA development than obese men [42], and women in the highest BMI tercile had six-fold increased risk of KOA development and 18-fold increased risk for bilateral KOA development compared to those in the lowest BMI tercile [43].

This study has some limitations. We could only use non-enhanced MRI images for the evaluation of effusion and Hoffa synovitis, which may not be an ideal way to measure synovitis. Case-control design of this study and matching may have a risk of selection bias. There may be a risk of having a collider bias in this study because it included subjects with radiographic KOA (KL grade ≥ 2) at baseline (Supplementary Table S2). Hence the effect of the risk factors of interest for radiographic KOA progression, including baseline BMI, might have been biased to the null. However, about 70% of the total subjects did not have radiographic KOA at baseline and the analysis of the sub-cohort that included only the subjects without radiographic KOA at baseline yielded similar results (data not shown). Given that most of the participants were white and that about one third of them had a high education level (graduate degree), generalizability of the findings of this study may be limited. Further studies among populations of more diverse socio-demographic backgrounds are needed. The risk of radiographic KOA progression could only be determined 2 years after baseline, when follow up MRI was performed. This necessitates further research about other potential biomarkers that might detect at-risk patients at the time point of baseline. Lastly, the participants.

## Conclusions

Based on these findings, radiographic KOA progression was associated with 24-month changes of IPFP size and SCAT. The increase in IPFP size over 24 months as well as the increase in SCAT, may be strong MRI markers for subsequent radiographic KOA progression.

### Electronic supplementary material

Below is the link to the electronic supplementary material.

Supplementary Material 1

Supplementary Material 2

Supplementary Material 3

Supplementary Material 4

## Data Availability

Data may be made available upon reasonable request after publication and after confirming that ethical approval has been obtained.

## Abbreviations

KOA: Knee osteoarthritis
IPFP: Infrapatellar fat pad
SCATthigh: Subcutaneous adipose tissue around distal thigh
KL score: Kellgren-Lawrence score
CSA: Cross sectional area
BMI: Body mass index
OR: Odds ratio
CI: Confidence interval
SD: Standard deviation
OA: Osteoarthritis
MRI: Magnetic resonance imaging
OAI: Osteoarthritis initiative
NIH: National institute of health
DESS: Dual echo in steady status
FLASH: Fast low-angle shot
MESE: Multi-echo spin-echo
MOAKS: MRI Osteoarthritis Knee Score
ANOVA: One way analysis of variance
IAAT: Intra-articular adipose tissue
