# Supplementary material for: Infrapatellar fat pad size and subcutaneous fat in knee osteoarthritis radiographic progression: data from the osteoarthritis initiative
Source: Arthritis Res Ther. 2024 Jul 30;26:145. doi: 10.1186/s13075-024-03367-w (PMC11289919; doi:10.1186/s13075-024-03367-w)
Supplement: Supplementary file 1 — Supplementary Material 1 [file 13075_2024_3367_MOESM1_ESM.docx]

|  | All (n=630) | Cases (n=315) | Controls (n=315) | *P* |
| --- | --- | --- | --- | --- |
| BMI (mean±SD, kg/m2) | 28.63±4.8 | 29.1±4.7 | 28.2±4.9 | 0.014 |
| BMI category |  |  |  |  |
| Normal weight (BMI<24.9 kg/m^2^, %) | 24.9 | 21 | 28.9 | 0.045 |
| Overweight (BMI 25-29.9 kg/m2, %) | 37.1 | 37.5 | 36.8 |  |
| Obesity (BMI ≥30 kg/m2, %) | 37.9 | 41.5 | 34.3 |  |
| Abdominal circumference (mean±SD, cm) | 102.43±12.80 | 103.91±12.83 | 100.95±12.62 | 0.004 |
| Height (mean±SD, cm) | 165.04±19.4 | 165.41±18.4 | 164.67±20.4 | 0.636 |
| Injury to the right knee (%) | 25.2 | 27.9 | 22.5 | 0.14 |
| Kellgren-Lawrence grade |  |  |  |  |
| Grade 0 (%) | 36.8 | 36.8 | 36.8 | 0.99 |
| Grade 1 (%) | 30.2 | 30.2 | 30.2 |  |
| Grade 2 (%) | 25.1 | 25.1 | 25.1 |  |
| Grade 3 (%) | 7.9 | 7.9 | 7.9 |  |
| Income |  |  |  |  |
| < $10K (%) | 2.4 | 2.3 | 2.4 | 0.97 |
| $10K to <$25K (%) | 8.3 | 9 | 7.6 |  |
| $25K to <$50K (%) | 25.3 | 25.4 | 25.1 |  |
| $50K to <$100K (%) | 40.0 | 39.8 | 40.2 |  |
| ≥$100K (%) | 24.1 | 23.5 | 24.7 |  |
| Diabetes Mellitus (%) | 5.1 | 4.4 | 5.7 | 0.59 |
| Education |  |  |  |  |
| High school graduate (%) | 11.5 | 12.2 | 11 | 0.50 |
| Some college (%) | 23.3 | 25.6 | 20.8 |  |
| College graduate (%) | 22.3 | 20.2 | 24.4 |  |
| Some graduate school (%) | 8.8 | 9.1 | 8.4 |  |
| Graduate degree (%) | 34.1 | 33 | 35.4 |  |

**Supplementary Table S1. Other risk factors of OA in the case and control group**

OA: osteoarthritis, BMI: body mass index, SD: standard deviation

**Supplementary Table S2A. Values of obesity and MRI markers at baseline per sex and BMI category**

| **Baseline values** | **Case (mean**±SD) | **Control (mean**±SD) | ***P*** |
| --- | --- | --- | --- |
| **BMI (kg/m^2^)** |  |  |  |
| All | 29.09±4.69 | 28.1±4.83 | 0.014 |
| Men | 28.75±3.71 | 28.62±3.88 | 0.815 |
| Women | 29.24±5.04 | 27.96±5.17 | 0.009 |
| Normal weight | 23.01±1.53 | 22.63±1.62 | 0.138 |
| Overweight | 27.44±1.41 | 27.48±1.48 | 0.834 |
| Obese | 33.65±2.84 | 33.53±2.91 | 0.761 |
| **Abdominal circumference (cm)** |  |  |  |
| All | 103.90±12.82 | 100.95±12.62 | 0.004 |
| Men | 104.57±11.03 | 104.07±10.91 | 0.761 |
| Women | 104.05±13.53 | 100.10±13.02 | 0.002 |
| Normal weight | 91.34±7.72 | 88.86±7.72 | 0.050 |
| Overweight | 100.27±9.39 | 99.95±7.96 | 0.783 |
| Obese | 113.48±10.21 | 112.31±9.69 | 0.370 |
| **IPFP CSA (cm^2^)** |  |  |  |
| All | 6.51±1.17 | 6.54±1.17 | 0.787 |
| Men | 7.50±1.12 | 7.52±1.10 | 0.891 |
| Women | 6.11±0.93 | 6.13±0.94 | 0.844 |
| Normal weight | 6.33±0.95 | 6.17±1.03 | 0.343 |
| Overweight | 6.51±1.24 | 6.56±1.09 | 0.738 |
| Obese | 6.61.201± | 6.82±1.29 | 0.188 |
| **SCAT*thigh* (cm^2^)** |  |  |  |
| All | 5.68±2.09 | 5.50±1.86 | 0.251 |
| Men | 3.73±1.21 | 3.71±1.09 | 0.911 |
| Women | 6.47±1.84 | 6.23±1.59 | 0.158 |
| Normal weight | 4.73±1.48 | 4.92±1.30 | 0.387 |
| Overweight | 5.32±1.87 | 5.30±1.85 | 0.939 |
| Obese | 6.47±2.25 | 6.19±2.05 | 0.308 |
| **Hoffa synovitis** |  |  |  |
| All | 0.78±0.72 | 0.44±0.54 | <0.001 |
| Men | 0.76±0.81 | 0.50±0.56 | 0.014 |
| Women | 0.78±0.69 | 0.42±0.34 | <0.001 |
| Normal weight | 0.74±0.64 | 0.43±0.54 | 0.001 |
| Overweight | 0.85±0.76 | 0.47±0.55 | <0.001 |
| Obese | 0.72±0.72 | 0.43±0.53 | <0.001 |
| **Effusion synovitis** |  |  |  |
| All | 0.71±0.67 | 0.41±0.51 | <0.001 |
| Men | 0.78±0.70 | 0.38±0.48 | <0.001 |
| Women | 0.68±0.66 | 0.42±0.52 | <0.001 |
| Normal weight | 0.71±0.70 | 0.42±0.53 | 0.004 |
| Overweight | 0.79±0.64 | 0.44±0.51 | <0.001 |
| Obese | 0.65±0.68 | 0.37±0.48 | <0.001 |

MRI: magnetic resonance imaging, BMI: body mass index, IPFP CSA: cross sectional area of infrapatellar fat pad, SCAT*thigh*: subcutaneous fat assessment around distal thigh

**Supplementary Table S2B. Values of obesity and MRI markers at 24 months per sex and BMI category**

| **Values at 24 months** | **Case (mean**±SD) | **Control (mean**±SD) | ***P*** |
| --- | --- | --- | --- |
| **BMI (kg/m^2^)** |  |  |  |
| All | 29.09±4.69 | 28.1±4.83 | 0.014 |
| Men | 28.81±3.84 | 28.39±3.90 | 0.469 |
| Women | 29.54±5.49 | 28.13±5.27 | 0.008 |
| Normal weight | 23.34±2.30 | 23.23±2.50 | 0.796 |
| Overweight | 27.54±1.90 | 27.49±1.86 | 0.847 |
| Obese | 33.93±3.63 | 33.39±3.53 | 0.266 |
| **Abdominal circumference (cm)** |  |  |  |
| All | 103.90±12.82 | 100.95±12.62 | 0.004 |
| Men | 103.54±11.00 | 102.99±11.40 | 0.736 |
| Women | 105.41±14.34 | 101.28±12.72 | 0.002 |
| Normal weight | 92.15±10.10 | 91.35±8.63 | 0.602 |
| Overweight | 101.73±9.01 | 101.11±8.19 | 0.596 |
| Obese | 114.95±10.8 | 112.75±9.51 | 0.112 |
| **IPFP CSA (cm^2^)** |  |  |  |
| All | 6.96±1.20 | 6.23±1.11 | <0.001 |
| Men | 8.04±1.15 | 7.12±1.08 | <0.001 |
| Women | 6.51±0.91 | 5.85±0.89 | <0.001 |
| Normal weight | 6.68±0.92 | 5.92±1.00 | <0.001 |
| Overweight | 6.92±1.32 | 6.32±1.05 | <0.001 |
| Obese | 7.13±1.21 | 6.43±1.22 | <0.001 |
| **SCAT*thigh* (cm^2^)** |  |  |  |
| All | 6.54±2.05 | 5.87±1.88 | <0.001 |
| Men | 4.40±1.16 | 4.01±0.89 | 0.016 |
| Women | 7.41±1.67 | 6.64±1.63 | <0.001 |
| Normal weight | 5.70±1.60 | 5.62±1.91 | 0.804 |
| Overweight | 6.09±1.80 | 5.63±1.79 | 0.066 |
| Obese | 7.38±2.18 | 6.39±1.89 | <0.001 |
| **Hoffa synovitis** |  |  |  |
| All | 0.78±0.72 | 0.44±0.54 | <0.001 |
| Men | 1.45±0.72 | 0.34±0.47 | <0.001 |
| Women | 1.17±0.70 | 0.31±0.47 | <0.001 |
| Normal weight | 1.15±0.63 | 0.34±0.50 | <0.001 |
| Overweight | 1.30±0.67 | 0.36±0.48 | <0.001 |
| Obese | 1.26±0.78 | 0.24±0.42 | <0.001 |
| **Effusion synovitis** |  |  |  |
| All | 0.71±0.67 | 0.41±0.51 | <0.001 |
| Men | 1.41±0.76 | 0.36±0.48 | <0.001 |
| Women | 1.39±0.73 | 0.37±0.49 | <0.001 |
| Normal weight | 1.27±0.71 | 0.44±0.52 | <0.001 |
| Overweight | 1.41±0.75 | 0.39±0.49 | <0.001 |
| Obese | 1.45±0.74 | 0.28±0.45 | <0.001 |

MRI: magnetic resonance imaging, BMI: body mass index, IPFP CSA: cross sectional area of infrapatellar fat pad, SCAT*thigh*: subcutaneous fat assessment around distal thigh

**Supplementary Table S2C. 24-month changes of obesity and MRI markers per sex and BMI category**

| **Changes over 24 months** | **Case (n=315, mean**±SD) | **Control (n=315, mean**±SD) | ***P*** |
| --- | --- | --- | --- |
| Δ **BMI (kg/m^2^)** |  |  |  |
| All | 0.22±1.76 | 0.14±1.95 | 0.633 |
| Men | 0.07±1.38 | -0.02±1.50 | 0.657 |
| Women | 0.28±1.90 | 0.22±2.11 | 0.752 |
| Normal weight | 0.24±1.76 | 0.58±1.88 | 0.253 |
| Overweight | 0.11±1.39 | 0.01±1.46 | 0.611 |
| Obese | 0.30±2.05 | -0.10±2.38 | 0.172 |
| Δ **Abdominal circumference (cm)** |  |  |  |
| All | 1.40±9.32 | 1.33±9.67 | 0.934 |
| Men | 1.23±5.52 | 1.53±6.62 | 0.740 |
| Women | 1.47±10.53 | 1.25±10.72 | 0.832 |
| Normal weight | 1.04±7.60 | 2.47±10.01 | 0.345 |
| Overweight | 1.18±9.29 | 1.05±9.20 | 0.922 |
| Obese | 1.78±10.16 | 0.62±9.87 | 0.392 |
| Δ **IPFP CSA (cm^2^)** |  |  |  |
| All | 0.47±0.54 | -0.27±0.43 | <0.001 |
| Men | 0.59±0.63 | -0.33±0.53 | <0.001 |
| Women | 0.43±0.50 | -0.25±0.39 | <0.001 |
| Normal weight | 0.35±0.51 | -0.28±0.42 | <0.001 |
| Overweight | 0.44±0.51 | -0.26±0.44 | <0.001 |
| Obese | 0.57±0.58 | -0.28±0.45 | <0.001 |
| Δ **SCAT*thigh* (cm^2^)** |  |  |  |
| All | 0.84±1.36 | 0.38±1.19 | <0.001 |
| Men | 0.70±1.37 | 0.25±1.00 | 0.02 |
| Women | 0.90±1.35 | 0.43±1.26 | <0.001 |
| Normal weight | 0.93±0.97 | 0.69±1.48 | 0.273 |
| Overweight | 0.80±1.27 | 0.35±1.07 | 0.006 |
| Obese | 0.84±1.59 | 0.12±0.95 | <0.001 |
| Δ **Hoffa synovitis** |  |  |  |
| All | 0.45±0.84 | -0.13±0.5 | <0.001 |
| Men | 0.69±0.89 | -0.15±0.50 | <0.001 |
| Women | 0.36±0.80 | -0.12±0.50 | <0.001 |
| Normal weight | 0.40±0.80 | -0.09±0.47 | <0.001 |
| Overweight | 0.40±0.81 | -0.11±0.50 | <0.001 |
| Obese | 0.53±0.89 | -0.19±0.51 | <0.001 |
| Δ **Effusion synovitis** |  |  |  |
| All | 0.67±0.77 | -0.06±0.52 | <0.001 |
| Men | 0.62±0.91 | -0.04±0.48 | <0.001 |
| Women | 0.69±0.71 | -0.07±0.53 | <0.001 |
| Normal weight | 0.56±0.74 | 0.01±0.52 | <0.001 |
| Overweight | 0.58±0.80 | -0.065±0.55 | <0.001 |
| Obese | 0.81±0.74 | -0.13±0.47 | <0.001 |

MRI: magnetic resonance imaging, BMI: body mass index, IPFP CSA: cross sectional area of infrapatellar fat pad, SCAT*thigh*: subcutaneous fat assessment around distal thigh, Δ: changes over 24 months from baseline

|  | **All** | **Case** | **Control** | **P** |
| --- | --- | --- | --- | --- |
| **BMI increase greater than 5% (%)** |  |  |  |  |
| All (n=593) | 103 (17.4) | 51 (17.2) | 52 (17.5) | 1.000 |
| Men (n=175) | 15 (8.6) | 7 (8.0) | 8 (9.1) | 1.000 |
| Women (n=418) | 88 (21.1) | 44 (21.1) | 44 (21.1) | 1.000 |
| Normal weight (n=152) | 31 (20.4) | 13 (20.6) | 18 (20.2) | 1.000 |
| Overweight (n=217) | 32 (14.7) | 14 (12.8) | 18 (16.7) | 0.450 |
| Obese (n=224) | 40 (17.9) | 24 (19.4) | 16 (16.0) | 0.600 |
| **Abdominal circumference increase greater than 5 cm (%)** |  |  |  |  |
| All (n=587) | 161 (27.4) | 76 (26.0) | 85 (28.8) | 0.461 |
| Men(n=175) | 38 (21.7) | 17 (19.5) | 21 (23.9) | 0.583 |
| Women (n=412) | 123 (29.9) | 59 (28.8) | 64 (30.9) | 0.667 |
| Normal weight (n=151) | 49 (32.5) | 18 (29.0) | 31 (34.8) | 0.484 |
| Overweight (n=216) | 48 (22.2) | 23 (21.1) | 25 (23.4) | 0.745 |
| Obese (n=220) | 64 (29.1) | 35 (28.9) | 29 (29.3) | 1.000 |
| **SCAT*thigh* increase greater than 1 SD (%)** |  |  |  |  |
| All (n=563) | 52 (9.2) | 39 (13.9) | 13 (4.6) | <0.001 |
| Men (n=164) | 8 (4.9) | 6 (7.4) | 2 (2.4) | 0.165 |
| Women (n=399) | 44 (11.0) | 33 (16.5) | 11 (5.5) | <0.001 |
| Normal weight (n=145) | 15 (10.3) | 8 (13.3) | 7 (8.2) | 0.409 |
| Overweight (n=212) | 13 (6.1) | 10 (9.5) | 3 (2.8) | 0.048 |
| Obese (n=206) | 24 (11.7) | 21 (18.1) | 3 (3.3) | <0.001 |
| **IPFP CSA increase greater than 1 SD (%)** |  |  |  |  |
| All (n=566) | 85 (15.0) | 83 (29.4) | 2 (0.7) | <0.001 |
| Men (n=167) | 34 (20.4) | 32 (39.0) | 2 (2.4) | <0.001 |
| Women (n=399) | 51 (12.8) | 51 (25.5) | 0 (0) | <0.001 |
| Normal weight (n=145) | 11 (7.6) | 11 (18.3) | 0 (0) | <0.001 |
| Overweight (n=212) | 31 (14.6) | 29 (27.6) | 2 (1.9) | <0.001 |
| Obese (n=209) | 43 (20.6) | 43 (36.8) | 0 (0) | <0.001 |
| **Hoffa synovitis worsened (%)** |  |  |  |  |
| All (n=566) | 146 (25.8) | 130 (46.1) | 16 (5.6) | <0.001 |
| Men (n=167) | 51 (30.5) | 47 (57.3) | 4 (4.7) | <0.001 |
| Women (n=399) | 95 (23.8) | 83 (41.5) | 12 (6.0) | <0.001 |
| Normal weight (n=145) | 34 (23.4) | 28 (46.7) | 6 (7.1) | <0.001 |
| Overweight (n=212) | 51 (24.1) | 45 (42.9) | 6 (5.6) | <0.001 |
| Obese (n=209) | 61 (29.2) | 57 (48.7) | 4 (4.3) | <0.001 |
| **Effusion synovitis worsened (%)** |  |  |  |  |
| All (n=566) | 192 (33.9) | 162 (57.4) | 30 (10.6) | <0.001 |
| Men (n=167) | 54 (32.3) | 46 (56.1) | 8 (9.4) | <0.001 |
| Women (n=399) | 138 (34.6) | 116 (58.0) | 22 (11.1) | <0.001 |
| Normal weight (n=145) | 43 (29.7) | 31 (51.7) | 12 (14.1) | <0.001 |
| Overweight (n=212) | 70 (33.0) | 57 (54.3) | 13 (12.1) | <0.001 |
| Obese (n=209) | 79 (37.8) | 74 (63.2) | 5 (5.4) | <0.001 |

**Supplementary Table S3. Changes of markers of obesity and synovitis over 24 months (dichotomous analysis) per sex and BMI**

BMI: body mass index, SD: standard deviation, IPFP CSA: cross sectional area of infrapatellar fat pad, SCAT*thigh*: subcutaneous fat assessment around distal thigh

|  | Men (n=184) | Women (n=446) |
| --- | --- | --- |
| Baseline BMI | 1.201 (0.790 – 1.826, P=0.390) | 1.303 (1.064 – 1.595, P=0.01) |
| Δ BMI | 1.129 (0.828 – 1.537, P=0.443) | 1.079 (0.879 – 1.324, P=0.468) |
| Baseline abdominal circumference | 1.131 (0.800 – 1.599, P=0.485) | 1.353 (1.107 – 1.653, P=0.003) |
| Δ Abdominal circumference | 0.989 (0.618 – 1.583, P=0.963) | 1.023 (0.860 – 1.218, P=0.797) |
| Baseline SCAT*thigh* | 0.933 (0.661 – 1.317, P=0.693) | 1.173 (0.954 – 1.443, P=0.130) |
| Δ SCAT*thigh* | 2.061 (1.130 – 3.760, P=0.018) | 1.562 (1.189 – 2.052, P=0.001) |
| Baseline IPFP CSA | 0.979 (0.699 – 1.372, P=0.904) | 0.996 (0.777 – 1.277, P=0.976) |
| Δ IPFP CSA | 6.032 (2.604 – 13.974, P<0.001) | 11.760 (5.735 – 24.118, P<0.001) |
| Baseline Hoffa synovitis | 1.384 (1.041 – 1.840, P=0.025) | 1.890 (1.494 – 2.391, P<0.001) |
| Δ Hoffa synovitis | 4.514 (2.176 – 9.366, P<0.001) | 2.275 (1.679 – 3.081, P<0.001) |
| Baseline effusion synovitis | 2.059 (1.391 – 3.048, P<0.001) | 1.598 (1.286 – 1.985, P<0.001) |
| Δ effusion synovitis | 2.678 (1.651 – 4.344, P<0.001) | 4.591 (2.930 – 7.193, P<0.001) |

**Supplementary Table S4. ORs for radiographic knee OA progression per sex**

OR: odds ratio, OA: osteoarthritis, BMI: body mass index, IPFP CSA: cross sectional area of infrapatellar fat pad, SCAT*thigh*: subcutaneous fat assessment around distal thigh, Δ: changes over 24 months from baseline

|  | Normal weight (n=157) | Overweight (n=234) | Obese (n=239) |
| --- | --- | --- | --- |
| Baseline abdominal circumference | 1.137 (0.585 – 2.211, P=0.704) | 1.000 (0.696 – 1.437, P=0.999) | 0.946 (0.645 – 1.389, P=0.778) |
| Δ Abdominal circumference | 0.951 (0.536 – 1.690, P=0.865) | 1.056 (0.740 – 1.507, P=0.763) | 1.342 (0.874 – 2.062, P=0.179) |
| Baseline SCAT*thigh* | 0.520 (0.179 – 1.515, P=0.231) | 0.979 (0.580 – 1.654, P=0.937) | 1.081 (0.635 – 1.841, P=0.774) |
| Δ SCAT*thigh* | 2.127 (0.797 – 5.681, P=0.132) | 2.409 (1.182 – 4.909, P=0.016) | 2.710 (1.238 – 5.930, P=0.013) |
| Baseline IPFP CSA | 1.239 (0.480 – 3.202, P=0.658) | 0.793 (0.467 – 1.348, P=0.392) | 0.725 (0.422 – 1.244, P=0.243) |
| Δ IPFP CSA | 22.887 (1.645 – 318.521, P=0.02) | 4.659 (1.894 – 11.457, P<0.001) | 81.436 (2.547 – 2603.519, P=0.013) |
| Baseline Hoffa synovitis | 2.298 (0.951 – 5.552, P=0.064) | 2.152 (1.293 – 3.583, P=0.003) | 1.730 (1.075 – 2.785, P=0.024) |
| Δ Hoffa synovitis | 2.810 (0.933 – 8.464, P=0.066) | 2.251 (1.192 – 4.252, P=0.012) | 3.854 (1.702 – 8.728, P=0.001) |
| Baseline effusion synovitis | 2.450 (1.042 – 5.764, P=0.04) | 1.934 (1.207 – 3.099, P=0.006) | 1.586 (1.018 – 2.471, P=0.041) |
| Δ effusion synovitis | 1.734 (0.824 – 3.651, P=0.147) | 3.302 (1.564 – 6.970, P=0.002) | 15.089 (2.531 – 89.958, P=0.003) |

**Supplementary Table S5. ORs for radiographic knee OA progression per baseline BMI category**

OR: odds ratio, BMI: body mass index, IPFP CSA: cross sectional area of infrapatellar fat pad, SCAT*thigh*: subcutaneous fat assessment around distal thigh, Δ: changes over 24 months from baseline

**Supplementary table S6A. Baseline values of obesity and imaging markers (incidence cohort)**

| **Baseline values** | **Case (n=95, mean±SD**) | **Control (n=327, mean±SD**) | ***P*** |
| --- | --- | --- | --- |
| BMI (kg/m^2^) | 29.29±4.78 | 27.85±4.75 | 0.010 |
| Abdominal circumference (cm) | 104.68±13.81 | 100.48±12.68 | 0.006 |
| IPFP CSA (cm^2^) | 6.63±1.20 | 6.65±1.19 | 0.859 |
| SCAT*thigh* (cm^2^) | 5.53±1.98 | 5.46±1.89 | 0.748 |
| Hoffa synovitis | 0.53±0.65 | 0.51±0.61 | 0.805 |
| Effusion synovitis | 0.51±0.60 | 0.44±0.55 | 0.309 |

**Supplementary table S6B. Changes over 24 months in obesity and imaging markers (incidence cohort)**

| **Values at 24 months** | **Case (n=87, mean**±SD) | **Control (n=306, mean**±SD) | ***P*** |
| --- | --- | --- | --- |
| BMI (kg/m^2^) | 29.51±4.82 | 27.97±4.92 | 0.010 |
| Abdominal circumference (cm) | 105.43±13.06 | 101.48±12.54 | 0.011 |
| IPFP CSA (cm^2^) | 7.04±1.20 | 6.54±1.22 | <0.001 |
| SCAT*thigh* (cm^2^) | 6.34±1.92 | 5.97±1.84 | 0.114 |
| Hoffa synovitis | 1.16±0.71 | 0.57±0.66 | <0.001 |
| Effusion synovitis | 1.34±0.77 | 0.61±0.69 | <0.001 |
| **Change over 24 months** | **Case (n=87, mean**±SD) | **Control (n=306, mean**±SD) | ***P*** |
| Δ BMI (kg/m^2^) | 0.20±1.70 | 0.19±1.70 | 0.931 |
| Δ Abdominal circumference (cm) | 1.03±7.14 | 1.07±9.64 | 0.970 |
| Δ IPFP CSA (cm^2^) | 0.44±0.53 | -0.06±0.60 | <0.001 |
| Δ SCAT*thigh* (cm^2^) | 0.78±1.53 | 0.51±1.09 | 0.077 |
| Δ Hoffa synovitis | 0.60±0.91 | 0.04±0.62 | <0.001 |
| Δ Effusion synovitis | 0.82±0.84 | 0.14±0.66 | <0.001 |

**Supplementary table S6C. Changes of markers of obesity and synovitis over 24 months (dichotomous analysis)**

|  | **All** | **Case** | **Control** | **P** |
| --- | --- | --- | --- | --- |
| BMI increase greater than 5% [n=393, n(%)] | 71 (18.1) | 16 (18.4) | 55 (18.0) | 0.929 |
| Abdominal circumference increased greater than 5 cm from baseline [n=390, n(%)] | 109 (27.9) | 20 (23.3) | 89 (29.3) | 0.341 |
| ΔSCAT*thigh* greater than 1 SD [n=374, n(%)] | 33 (8.8) | 12 (14.3) | 21 (7.2) | 0.051 |
| ΔIPFP CSA greater than 1 SD [n=376, n(%)] | 50 (13.3) | 21 (24.7) | 29 (10.0) | <0.001 |
| Hoffa synovitis worsened [n=376, n(%)] | 96 (25.5) | 48 (56.5) | 48 (16.5) | <0.001 |
| Effusion synovitis worsened [n=376, n(%)] | 124 (33.0) | 55 (64.7) | 69 (23.7) | <0.001 |

**Supplementary table S6D. ORs for incident KOA (incident cohort)**

|  | *OR (95% CI) | *P* |
| --- | --- | --- |
| Baseline BMI | 1.617 (1.142 – 2.288) | 0.007 |
| Δ BMI | 1.165 (0.806 – 1.682) | 0.565 |
| Baseline abdominal circumference | 1.442 (1.059 – 1.962) | 0.020 |
| Δ abdominal circumference | 1.291 (0.849 – 1.291) | 0.232 |
| Baseline SCAT*thigh* | 1.379 (0.888 – 2.139) | 0.152 |
| Δ SCAT*thigh* | 1.578 (1.032 – 2.412) | 0.035 |
| Baseline IPFP CSA | 0.954 (0.677 – 1.345) | 0.788 |
| Δ IPFP CSA | 6.855 (2.897 – 16.220) | <0.001 |
| Baseline Hoffa’s synovitis | 1.319 (0.965 – 1.803) | 0.082 |
| Δ Hoffa’s synovitis | 3.690 (2.023 – 6.731) | <0.001 |
| Baseline effusion synovitis | 1.394 (0.979 – 1.983) | 0.065 |
| Δ Effusion synovitis | 5.187 (2.459 – 10.943) | <0.001 |

**Supplementary table S6E. Mediation analysis of the effect of baseline BMI on incident KOA mediated by IPFP CSA change over 2 years (Incident cohort)**

| Independent variable (X) | Indirect effect Coefficient (95% CI) | Direct effect Coefficient (95% CI) | Mediation (%) |
| --- | --- | --- | --- |
| *All (n=422)* |  |  |  |
| Baseline BMI | 0.0177 (0.009, 0.0388) | 0.0456 (-0.0092, 0.1005) | 27.9 |
| *Men only (n=132)* |  |  |  |
| Baseline BMI | 0.0235 (-0.0196, 0.0820) | 0.0813 (-0.0356, 0.1982) | 22.4 |
| *Women only (n=290)* |  |  |  |
| Baseline BMI | 0.0167 (-0.0026, 0.0406) | 0.0366 (-0.0266, 0.0998) | 31.3 |

**Supplementary table S7A. Baseline values of obesity and imaging markers (progression cohort)**

| **Baseline values** | **Case (n=211, mean±SD**) | **Control (n=211, mean±SD**) | ***P*** |
| --- | --- | --- | --- |
| BMI (kg/m^2^) | 28.67±4.58 | 27.68±4.96 | 0.034 |
| IPFP CSA (cm^2^) | 6.65±1.21 | 6.65±1.17 | 0.998 |
| SCAT*thigh* (cm^2^) | 5.61±2.01 | 5.34±1.79 | 0.142 |
| Abdominal circumference (cm) | 103.03±12.95 | 99.83±12.98 | 0.012 |
| Hoffa synovitis | 0.65±0.68 | 0.39±0.52 | <0.001 |
| Effusion synovitis | 0.58±0.62 | 0.33±0.47 | <0.001 |

**Supplementary table S7B. Changes over 24 months in obesity and imaging markers (progression cohort)**

| **Values at 24 months** | **Case (n=102, mean**±SD) | **Control (n=98, mean**±SD) | ***P*** |
| --- | --- | --- | --- |
| BMI (kg/m^2^) | 30.02±5.37 | 29.28±4.66 | 0.303 |
| IPFP CSA (cm^2^) | 6.79±1.18 | 6.17±1.09 | <0.001 |
| SCAT*thigh* (cm^2^) | 6.71±2.28 | 6.29±2.13 | 0.093 |
| Abdominal circumference (cm) | 106.70±14.19 | 105.63±11.60 | 0.563 |
| Hoffa synovitis | 1.45±0.78 | 0.43±0.51 | <0.001 |
| Effusion synovitis | 1.65±0.72 | 0.54±0.52 | <0.001 |
| **Change over 24 months** | **Case (n=102, mean**±SD) | **Control (n=98, mean**±SD) | ***P*** |
| Δ BMI (kg/m^2^) | 0.17±2.10 | 0.26±2.19 | 0.523 |
| Δ IPFP CSA (cm^2^) | 0.53±0.55 | -0.15±0.42 | <0.001 |
| Δ SCAT*thigh* (cm^2^) | 0.92±1.34 | 0.46±1.56 | 0.031 |
| Δ Abdominal circumference (cm) | 1.31±10.09 | 2.64±10.21 | 0.360 |
| Δ Hoffa synovitis | 0.42±0.90 | -0.14±0.52 | <0.001 |
| Δ Effusion synovitis | 0.67±0.72 | -0.05±0.57 | <0.001 |

**Supplementary table S7C. Changes of markers of obesity and synovitis over 24 months (dichotomous analysis)**

|  | **All** | **Case** | **Control** | **P** |
| --- | --- | --- | --- | --- |
| BMI increase greater than 5% [n=200, n(%)] | 32 (16.0) | 12 (11.8) | 20 (20.4) | 0.123 |
| Abdominal circumference increased greater than 5 cm from baseline [n=197, n(%)] | 52 (26.4) | 21 (21.0) | 31 (32.0) | 0.106 |
| ΔSCAT*thigh* greater than 1 SD [n=189, n(%)] | 19 (10.1) | 13 (13.7) | 6 (6.4) | 0.145 |
| ΔIPFP CSA greater than 1 SD [n=190, n(%)] | 35 (18.4) | 35 (36.8) | 0 (0.0) | <0.001 |
| Hoffa synovitis worsened [n=190, n(%)] | 50 (26.3) | 44 (46.3) | 6 (6.3) | <0.001 |
| Effusion synovitis worsened [n=190, n(%)] | 68 (35.8) | 55 (57.9) | 13 (13.7) | <0.001 |

**Supplementary table S7D. ORs for radiographic KOA progression (progression cohort)**

|  | *OR (95% CI) | *P* |
| --- | --- | --- |
| Baseline BMI | 1.302 (0.941 – 1.801) | 0.112 |
| Δ BMI | 0.907 (0.705 – 1.167) | 0.448 |
| Baseline abdominal circumference | 1.280 (0.948 – 1.730) | 0.107 |
| Δ abdominal circumference | 0.906 (0.704 – 1.165) | 0.440 |
| Baseline SCAT*thigh* | 0.994 (0.710 – 1.392) | 0.972 |
| Δ SCAT*thigh* | 1.415 (0.960 – 2.088) | 0.080 |
| Baseline IPFP CSA | 0.889 (0.615 – 1.285) | 0.531 |
| Δ IPFP CSA | 10.576 (3.734 – 29.954) | <0.001 |
| Baseline Hoffa’s synovitis | 2.028 (1.452 – 2.833) | <0.001 |
| Δ Hoffa’s synovitis | 2.166 (1.451 – 3.234) | <0.001 |
| Baseline effusion synovitis | 1.853 (1.337 – 2.569) | <0.001 |
| Δ Effusion synovitis | 4.994 (2.409 – 10.354) | <0.001 |

**Supplementary table S7E. Mediation analysis of the effect of baseline BMI on radiographic KOA progression mediated by IPFP CSA change over 2 years (progression cohort)**

| Independent variable (X) | Indirect effect Coefficient (95% CI) | Direct effect Coefficient (95% CI) | Mediation (%) |
| --- | --- | --- | --- |
| *All (n=208)* |  |  |  |
| Baseline BMI | 0.0971 (0.0390, 0.1813) | -0.0120 (-0.0886, 0.0645) |  |
| *Men only (n=132)* |  |  |  |
| Baseline BMI | 0.0685 (-0.1426, 0.4408) | -0.1312 (-0.3863, 0.1240) |  |
| *Women only (n=156)* |  |  |  |
| Baseline BMI | 0.1029 (0.0399, 0.2021) | 0.0031 (-0.0793, 0.0855) | 97.7 |

**Supplementary Figure S1. Cross sectional area of IPFP and subcutaneous fat thickness measurements. A)** Mid-sagittal intermediate-weighted image of the knee demonstrating the method for quantifying the maximal cross-sectional area of the infrapatellar fat pad. **B)** Axial MPR reconstruction of an intermediate-weighted image of the distal thigh, just above the level of the knee, demonstrating the method of subcutaneous fat thickness quantification. Firstly, the thigh is divided into four quadrants. Then, the maximal thickness of the subcutaneous fat in each quadrant is measured as the maximal distance between the skin and the deep fascia.

**Supplementary Figure S2. Illustration of the concepts of the mediation analysis**
