# Supplementary figures and images for: Infrapatellar fat pad size and subcutaneous fat in knee osteoarthritis radiographic progression: data from the osteoarthritis initiative
Source: Arthritis Res Ther. 2024 Jul 30;26:145. doi: 10.1186/s13075-024-03367-w (PMC11289919; doi:10.1186/s13075-024-03367-w)

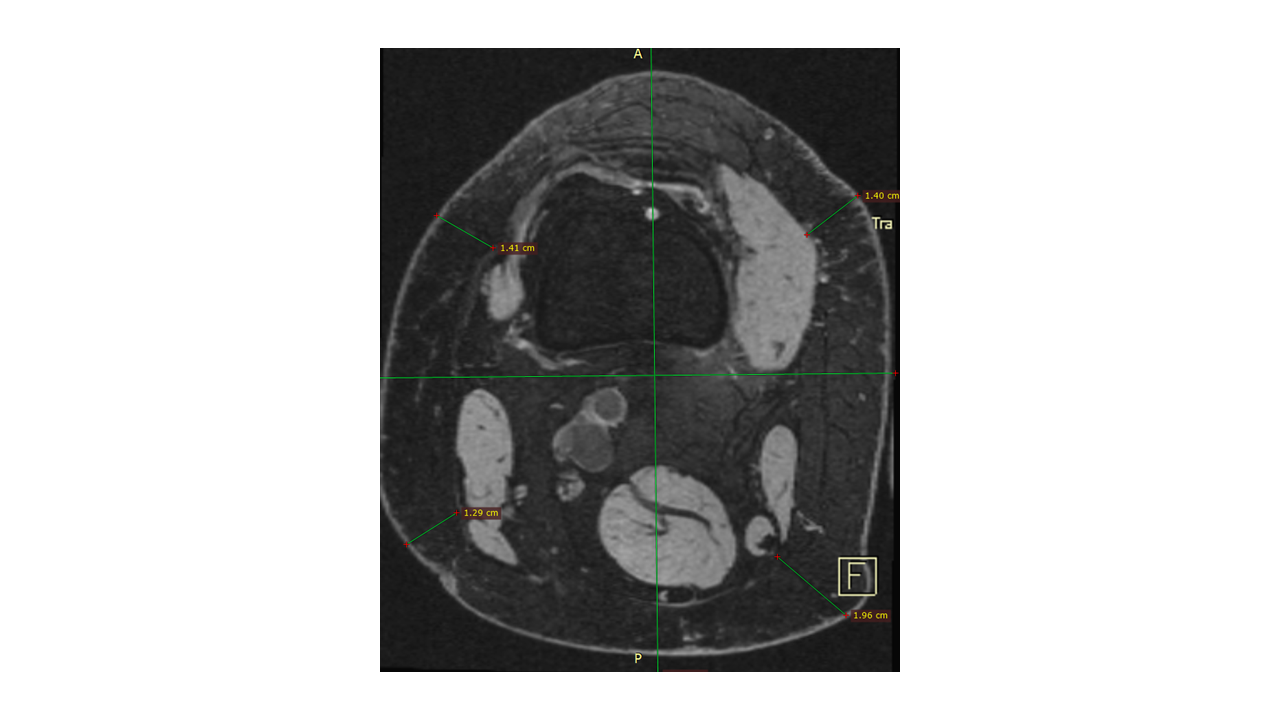

Supplement: Supplementary file 2 — Supplementary Material 2 [file 13075_2024_3367_MOESM2_ESM.tif]

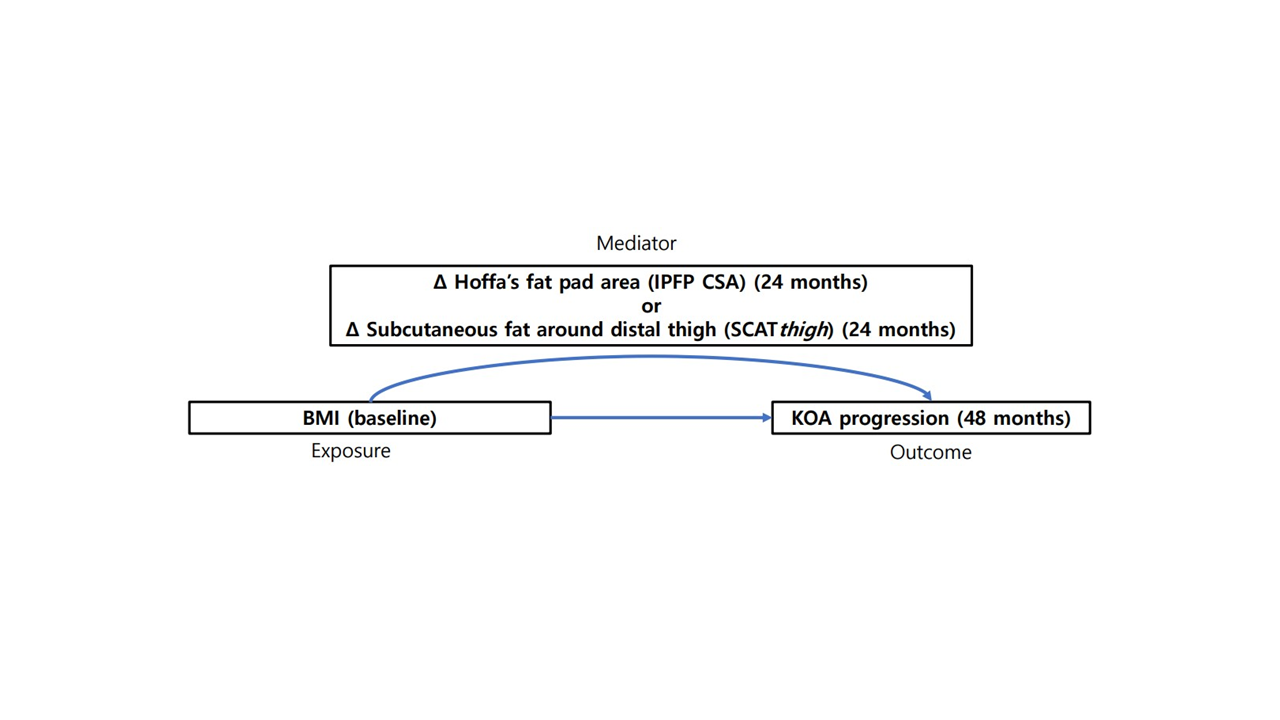

Supplement: Supplementary file 3 — Supplementary Material 3 [file 13075_2024_3367_MOESM3_ESM.tif]

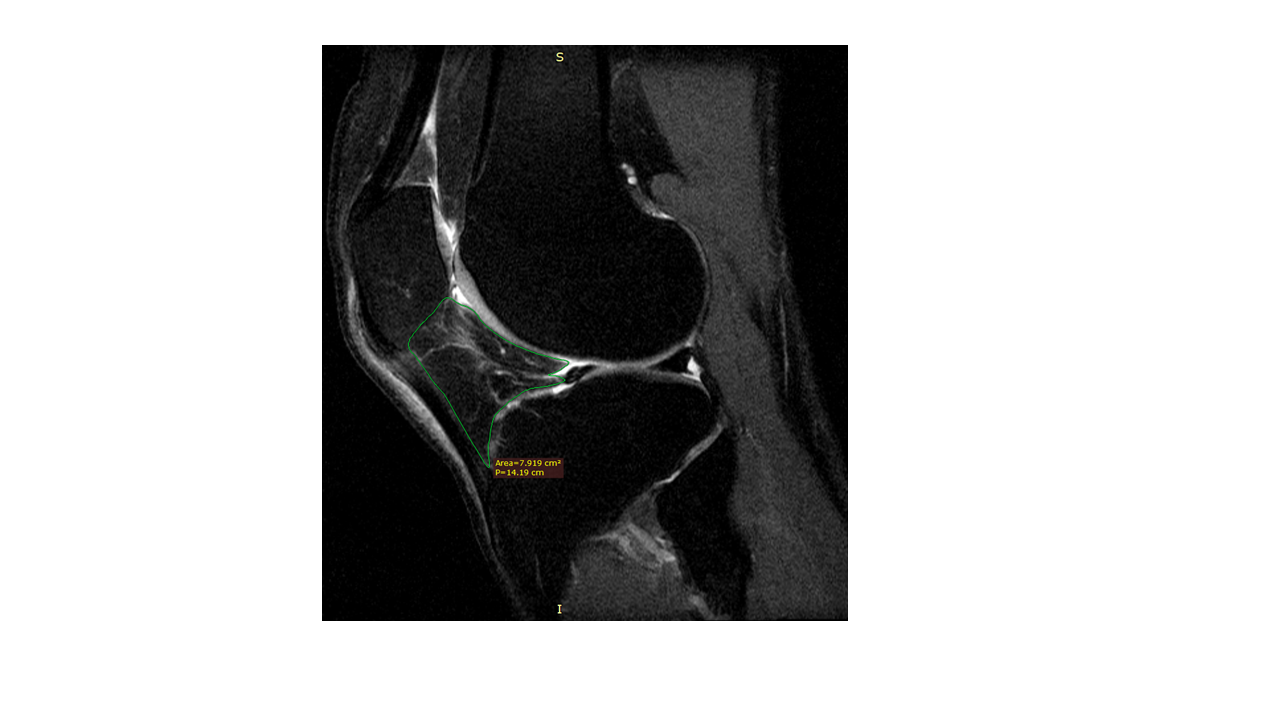

Supplement: Supplementary file 4 — Supplementary Material 4 [file 13075_2024_3367_MOESM4_ESM.tif]
